# Supplementary figures and images for: Translation, reliability, and validity of Japanese version of the Respiratory Distress Observation Scale
Source: PLoS One. 2021 Aug 11;16(8):e0255991. doi: 10.1371/journal.pone.0255991 (PMC8357131; doi:10.1371/journal.pone.0255991)

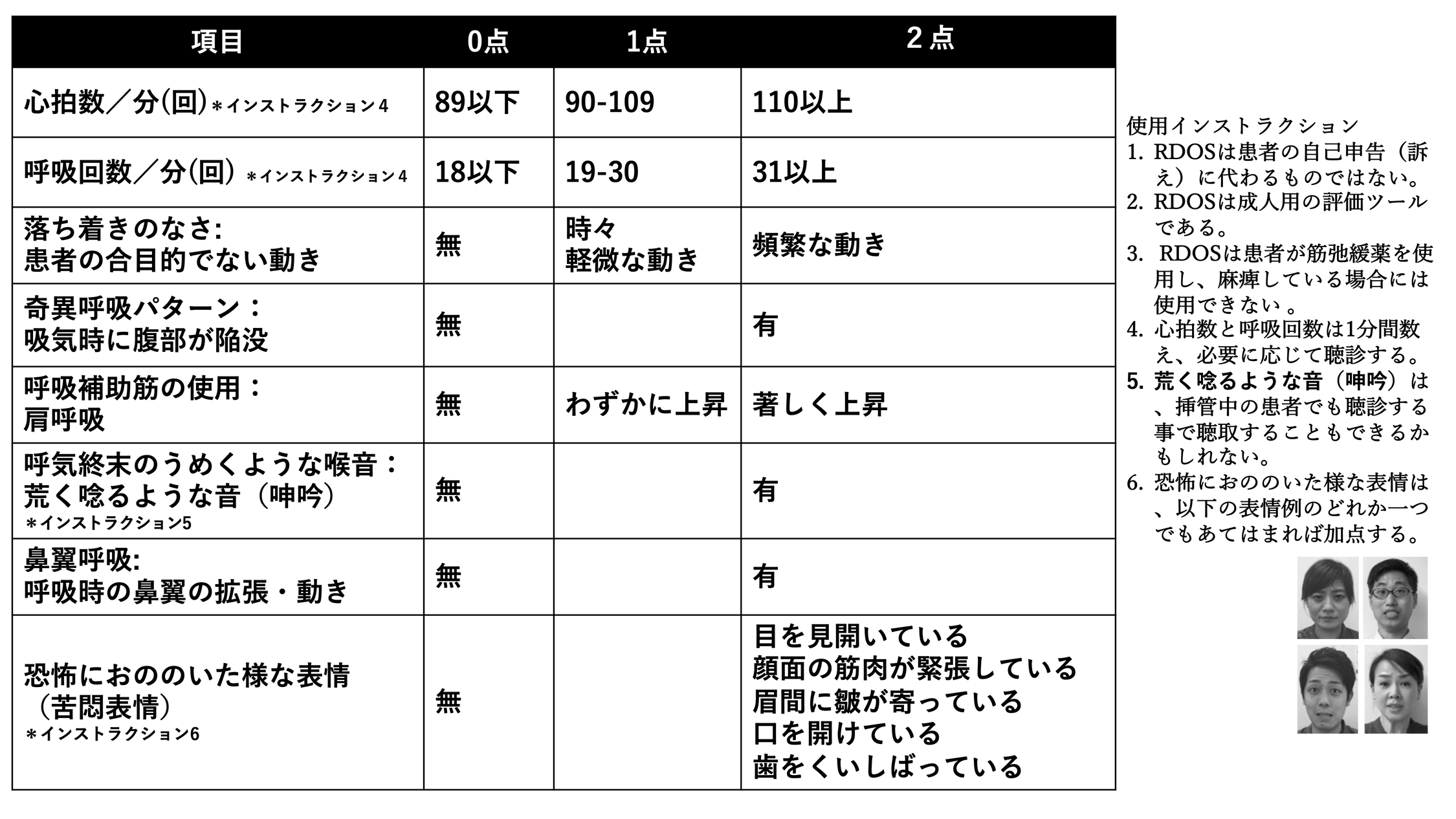

Supplement: S1 Fig — (TIF) [file pone.0255991.s001.tif]
